# Anti-amyloid antibody equilibrium binding to Aβ aggregates from human Alzheimer disease brain

**DOI:** 10.1101/2025.05.20.654902

**Published:** 2025-05-21

**Authors:** P. Monroe Butler, Anna Francis, Angela L. Meunier, Amirah K. Anderson, Elizabeth L. Hennessey, Michael B. Miller, Cynthia A. Lemere, Dennis J. Selkoe, Andrew M. Stern

**Affiliations:** 1. Ann Romney Center for Neurologic Diseases, Department of Neurology, Brigham and Women’s Hospital, Harvard Medical School, Boston, MA; 2. Department of Pathology, Brigham and Women’s Hospital, Harvard Medical School, Boston, MA

## Abstract

**Importance:**

Anti-amyloid immunotherapy is used to treat Alzheimer disease (AD) with moderate benefits and potentially serious side effects due to amyloid related imaging abnormality with effusions/edema (ARIA-E). Different anti-amyloid antibodies have different *in vitro* binding characteristics to different synthetic Aβ aggregates, leading to the assumption that they bind different species in the human brain. Lecanemab is hypothesized to bind “protofibrils,” but these are not well-characterized in human brain. It is also unknown how binding differences correlate with ARIA-E rates. The *APOE ε4* allele increases ARIA-E risk, but how it affects antibody binding characteristics is unknown.

**Objectives:**

To determine whether anti-amyloid antibodies bind different species of human brain Aβ and whether these binding properties to human brain Aβ explains ARIA-E rates.

**Design:**

Cross-sectional study of 18 postmortem human brains.

**Setting:**

Single tertiary care hospital.

**Participants:**

Deceased patients with AD and cerebral amyloid angiopathy (CAA).

**Main Outcomes and Measures:**

Equilibrium binding constants (K_D_) and total Aβ binding (B_max_) of recombinant aducanumab, lecanemab, and donanemab equivalents to human brain soluble and insoluble amyloid plaque-enriched and CAA-enriched Aβ aggregates.

**Results:**

Lecanemab did not bind with greater affinity to the soluble fraction of Aβ compared to aducanumab. All three antibodies were bound essentially identical quantities of Aβ across the 18 cases and fractions (Pearson’s r 0.84 – 0.97). Antibody preference for plaque *vs* CAA Aβ did not differ in soluble fractions but differed slightly in insoluble extracts. The *APOE ε4* allele led to a more soluble antibody-accessible Aβ pool in a dose-dependent manner for all three antibodies.

**Conclusions and Relevance:**

The lecanemab binding target in human brain is unlikely to be distinctly “protofibrillar” compared to other antibodies. Differences in antibody preference for plaque *vs* CAA Aβ are unlikely to fully explain differences in ARIA-E rates. The *APOE ε4* allele may plausibly increase ARIA-E risk by making antibody-accessible Aβ more soluble. These results have implications for improving the safety and efficacy of current and future anti-amyloid antibody therapies.

## Introduction

The introduction of disease-modifying therapy is changing the standard of care for Alzheimer disease. Three anti-amyloid antibodies, aducanumab, lecanemab, and donanemab, have received United States Food and Drug Administration approval to treat early Alzheimer disease (AD). Lecanemab and donanemab have entered clinical use. All three antibodies target misfolded, aggregated Aβ, the principal component of amyloid plaques. All three clear amyloid plaques as measured by amyloid positron emission tomography (PET) and avoid binding to monomeric Aβ. However, all three are thought to derive their preference for misfolded, aggregated Aβ over monomeric Aβ through different mechanisms. Donanemab binds to pyroglutamate-3 Aβ, a posttranslational modification found in plaques but not newly synthesized Aβ monomers. Lecanemab and aducanumab both bind conformational epitopes of misfolded Aβ. Between lecanemab and aducanumab, lecanemab is purported to have greater specificity for toxic protofibrils, rather than “mature” amyloid fibrils, and this distinction is postulated to account for its better observed efficacy^1^. However, binding preferences of all three antibodies have been published almost entirely using synthetic *in vitro* Aβ assemblies, rather than Aβ aggregates derived from human brain tissue. Structures of *in vitro* amyloids may differ from *in vivo* ones, and binding properties to *in vitro* assemblies do not infer binding properties to those present in the human brain.

All plaque-clearing anti-amyloid antibodies cause amyloid related imaging abnormality with edema/effusions (ARIA-E), a side effect that can rarely be fatal, and the cause of ARIA-E is not currently known. One hypothesis for the cause of ARIA-E is inflammation and blood-brain barrier breakdown due to binding of anti-amyloid antibodies to cerebral amyloid angiopathy (CAA), Aβ aggregates in the tunica media of brain arterioles and small arteries instead of (or in addition to) in the neuropil^2^. Support for this hypothesis comes from the observations that MRI-detectable lobar microhemorrhages, usually caused by CAA in the AD population, increase risk for ARIA-E; that ARIA-E exhibits clinical and radiographic similarity to CAA-related inflammation (CAAri), a sporadic disease possibly mediated by endogenous anti-Aβ autoantibodies; and autopsies of rare fatal ARIA-E cases have demonstrated vasculitis^3,4^.

These observations can be compiled into a theory in which 1) lecanemab, aducanumab, and donanemab bind structurally different classes of Aβ aggregates in human AD brain, and 2) differences in ARIA-E rates (aducanumab > donanemab > lecanemab) are attributable to differences in binding preference to CAA Aβ vs plaque Aβ. In the current study, we sought to test predictions of this theory: 1) certain populations of Aβ aggregates from human AD brain (*i.e.* “protofibrils”) will be accessible to binding to lecanemab but not aducanumab or donanemab, 2) lecanemab will exhibit greater binding affinity to aqueously extracted (“soluble,” “protofibrillar”) Aβ aggregates than aducanumab, and 3) binding preference of all three antibodies for CAA vs plaque Aβ will follow the inverse rank-order of observed ARIA-E rates in phase 3 clinical trials.

## Methods

### Case selection

We selected eighteen cases from the BWH Brain Bank with pathological diagnoses of both AD and CAA as described in Table 1. As expected for a population enriched for both AD and CAA, the *APOE* genotypes were enriched for the *ε4* allele. We extracted Aβ in two ways as described in detail in the Supplementary Information: 1) an aqueous extraction composed of material in the supernatant after homogenization in TBS followed by centrifugation, and 2) an insoluble extraction from the pellet of the first extraction. Separate extracts were prepared from the grey matter of the occipital lobe and from the overlying leptomeninges.

### Measurement of equilibrium binding affinity

**Figure 1.**
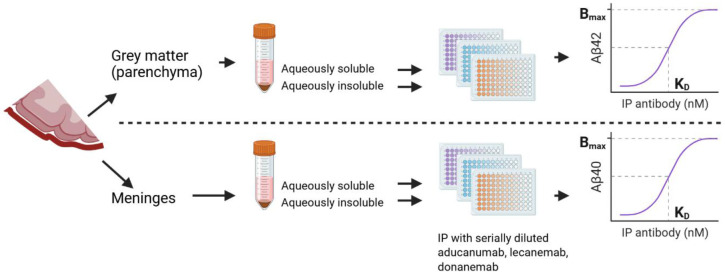
Experimental overview. Occipital lobe grey matter and overlying meninges from 18 cases with AD and CAA were processed to extract aqueously soluble and insoluble fractions. These were immunoprecipitated with serially diluted anti-amyloid antibodies followed by wash, elution, denaturation into monomers, and quantitation of total Aβ_42_ (for parenchyma) or Aβ_40_ (for meninges). Binding curves were fit to a one-site specific model and generated a K_D_, expressed in nM antibody and approximating equilibrium binding affinity, and a B_max_, expressed in ng/ml Aβ and reflecting the total amount of Aβ accessible to the antibody.

A full description of the biochemical methods, rationale, and validation is available in the Supplementary Information. Briefly, liquid extracts (aqueously soluble or insoluble, from parenchyma or from meninges) were diluted and immunoprecipitated with serially diluted anti-amyloid antibody on magnetic beads, followed by washing and ELISA quantitation of Aβ42 and Aβ40. The resulting titration curves were fitted to a one-site binding model resulting in a K_D_ (approximation of binding affinity) and B_max_ (total Aβ available to the antibody to bind) in GraphPad Prism software (Fig 1).

### Statistics

For our primary statistical analyses, we used the ratios of meningeal:parenchymal or insoluble:aqueous K_D_s and B_max_s as dependent variables. Because different antibody preparations can have different degrees of degradation or inactivity, normalizing by within-antibody ratios avoids this artifact. Expressing the K_D_ as a ratio can be interpreted as an antibody’s preference for one type of Aβ over another (*e.g.* soluble *vs* insoluble). We treated the unadjusted K_D_ and B_max_ without ratios as secondary outcomes. We refer to the meningeal:parenchymal ratios as “MP ratio” and the insoluble:soluble ratio as “IS ratio.”

All ratios were log-transformed for statistical analyses. We used a linear mixed model of the log(ratio) as the dependent variable, the random effect of patient (case), and the fixed effects of age, sex, antibody, and number of *APOE ε4* alleles. In all cases, the mixed models were fit by restricted maximum likelihood. T-tests used Satterthwaite’s method setting alpha to 0.05. Statistics were performed using R package lme4.

## Results

### Lecanemab does not exhibit greater preference for aqueously soluble (“protofibrillar”) Aβ compared to aducanumab

**Figure 2.**
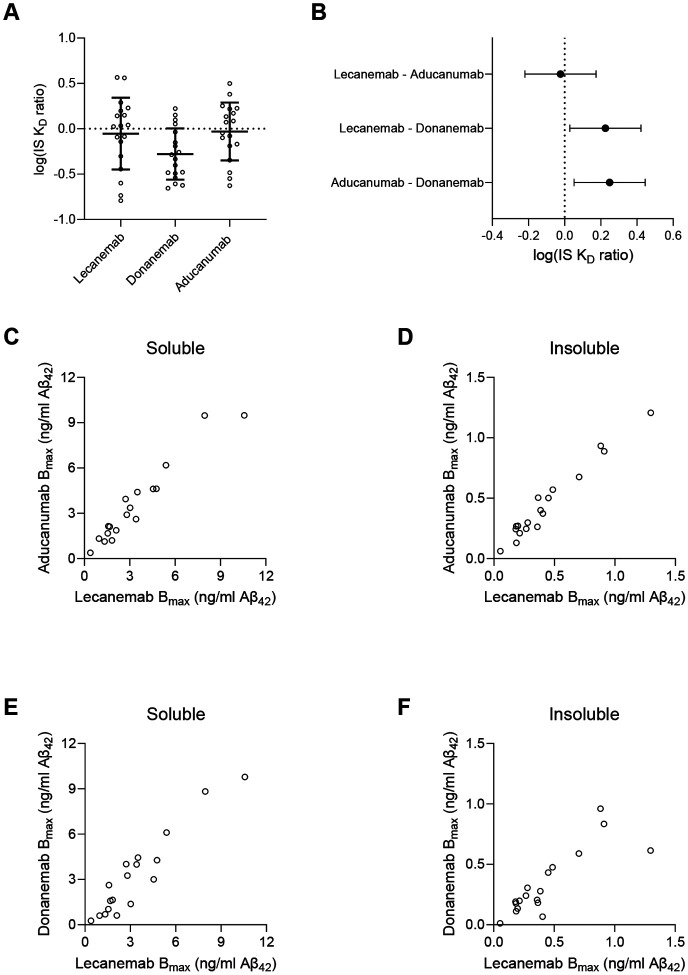
Binding profiles to insoluble *vs* soluble parenchymal Aβ_42_ aggregates. **(A)** The log(IS K_D_ ratio) reflects the binding preference of antibodies to insoluble *vs* soluble aggregates. A higher ratio implies greater preference for soluble aggregates. Donanemab exhibited a log ratio below 0, reflecting a slight preference for insoluble aggregates. Error bars = mean +/− SD. (**B)** Model estimates for the pairwise mean differences +/− 95% CI in log(IS K_D_ ratio). There was no statistically significant difference between lecanemab and aducanumab, but donanemab had a statistically different ratio compared to lecanemab and aducanumab. (**C-F)** Correlations between B_max_, the total Aβ accessible to the antibody, across soluble and insoluble extracts reveal near-perfect correlations.

The definition of Aβ protofibrils extracted from AD brain has been those which remain in the supernatant after ultracentrifugation in aqueous buffer^5-9^. If lecanemab had greater preference for protofibrils than fibrils compared to other antibodies, then one would expect a higher ratio of its K_D_ for insoluble to soluble aggregates (IS K_D_ ratio) compared to other antibodies, in particular for parenchymal samples, where all the intended antibody targets lie. However, we detected no statistically significant difference between the lecanemab and aducanumab parenchymal IS K_D_ ratios (Fig 2A). The 95% confidence interval for the difference between lecanemab and aducanumab log(IS K_D_ ratios) was −0.220 to 0.174, meaning our results are 95% confident that lecanemab has between a 1.66-fold lesser and 1.49-fold greater preference for aqueously extracted Aβ (*i.e.* protofibrils) than aducanumab (Fig 2B). Thus, we conclude that lecanemab is unlikely to have greater preference for aqueously soluble Aβ aggregates than does aducanumab. However, our results did suggest that lecanemab and aducanumab have greater preference for aqueously extracted Aβ compared to donanemab (lecanemab 95% CI 0.029 to 0.422, 1.07-fold to 2.64-fold; aducanumab 95% CI 0.052 to 0.445, 1.12- to 2.79-fold) (Fig. 2B). Full model results presented in the supplementary tables.

### Lecanemab and aducanumab access the same pool of Aβ aggregates

The B_max_, the amount of Aβ immunoprecipitated at saturating antibody concentrations, is a measure of the total Aβ in a brain extract accessible to an antibody. If lecanemab bound a distinct pool of Aβ aggregates to which aducanumab or donanemab could not bind, then one might expect the B_max_ of lecanemab to exceed that of the others. Alternatively, if different AD patients’ brains contained different amounts of protofibrillar *vs.* fibrillar Aβ, then there would be an imperfect correlation between B_max_s of different antibodies across different cases. However, we found a perfect 1:1 correlation of lecanemab and aducanumab B_max_ in both the aqueously soluble and insoluble fractions (r = 0.97, P = 6.5E-11) (Fig 2C, D). The donanemab B_max_ also correlated with the B_max_ of lecanemab for soluble and insoluble fractions albeit with slightly weaker correlations (soluble r = 0.94, P = 4.5E-9; insoluble r = 0.84, p = 1.1E-5, Fig 2F). Overall, we conclude that all three antibodies access essentially identical populations of aggregates. Donanemab may bind only a subset of pyroglutamate-3 epitopes, but we reason that these are distributed evenly enough among the Aβ aggregates that all aggregates can be bound by donanemab at least one site.

### Antibody binding preference does not explain ARIA-E rates in clinical trials

**Fig 3.**
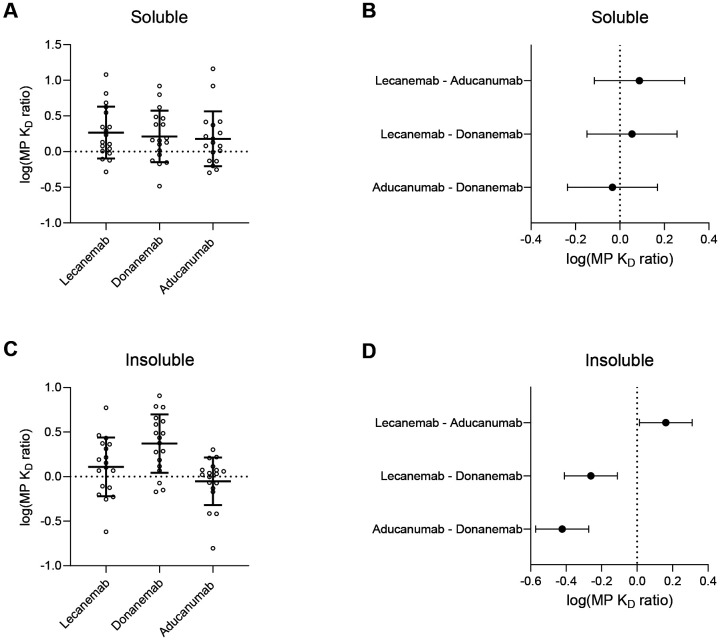
Plaque *vs* CAA antibody binding preferences. **(A, C)** The log(MP K_D_ ratio) reflects binding preferences of antibodies to meningeal Aβ_40_-rich aggregates (CAA-enriched) *vs* parenchymal Aβ_42_-rich aggregates (plaque-enriched). A higher ratio implies greater preference for plaque *vs* CAA aggregates. Error bars = mean +/− SD. **(B, D)** Model estimates for the pairwise mean differences +/− 95% CI in log(MP K_D_ ratio) reveals no significant differences in the soluble fraction but significant differences in the insoluble fraction. The difference in insoluble log(MP K_D_ ratio) between lecanemab and aducanumab reflects a 1.03- to 2.05-fold greater lecanemab preference for plaque compared to aducanumab, less than the ~2.8-fold difference in phase 3 clinical trials.

In phase 3 trials, aducanumab caused ARIA-E in 35.2% of subjects receiving drug^10^, followed by 24.0% by donanemab^11^ and 12.6% by lecanemab^12^. If the differences in ARIA-E rates were due to differences in binding preferences for CAA vs plaque Aβ, then one would expect the ratio of K_D_ for meningeal Aβ_40_-rich to parenchymal Aβ_42_-rich aggregates (MP K_D_ ratio) to differ among the three antibodies and follow the order lecanemab > donanemab > aducanumab. We detected no statistically significant differences among the MP K_D_ ratios of the three antibodies in the soluble pool (Fig 3A). The 95% confidence interval for the difference in MP K_D_ ratio of lecanemab *vs.* aducanumab in the soluble pool was −0.115 to 0.291 (Fig 3B). Thus we conclude that in aqueous extracts, lecanemab has between a 1.30-fold lesser and a 1.95-fold greater preference for plaque *vs* CAA fibrils compared to aducanumab, unlikely to explain fully the ~2.8-fold difference in ARIA-E rates in phase 3 clinical trials. Full model results are presented in the supplementary tables.

In insoluble extracts, there were statistically significant differences in MP K_D_ ratio, including all three pairwise comparisons (Fig 3C). However, the rank order was donanemab > lecanemab > aducanumab, as opposed to the expected order lecanemab > donanemab > aducanumab based on phase 3 trial ARIA results. Examining only lecanemab and aducanumab, which do exhibit the hypothesized order based on trial results (lecanemab>aducanumab), the 95% confidence interval for the difference in MP K_D_ ratio was 0.013 to 0.311 (Fig 3D), meaning lecanemab exhibited between a 1.03- and 2.05-fold greater preference for plaque *vs* CAA fibrils compared to aducanumab. Although this difference is statistically significant, it may not fully explain the observed nearly 3-fold difference in ARIA rates, nor account for donanemab having still greater preference for plaque compared to lecanemab or aducanumab. Full model results are presented in the supplementary tables.

### Effect of *APOE* genotype on antibody binding

**Fig 4.**
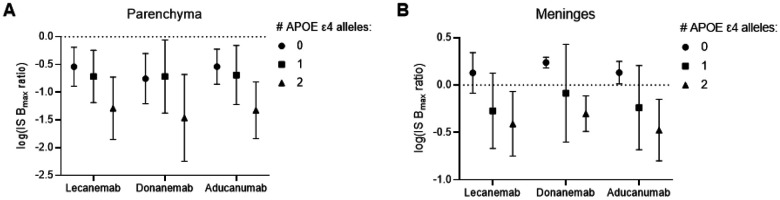
*APOE* genotype effects on Aβ accessible to antibody binding. The log(IS B_max_ ratio) reflects the solubility of the Aβ pool accessible to the antibody. A higher log(IS B_max_ ratio) reflects a less soluble pool of Aβ. We found that the *APOE ε4* dosage increased the solubility (decreased the log(IS B_max_ ratio)) of both parenchymal Aβ_42_ **(A)** and meningeal Aβ_40_ antibody targets. Error bars = mean +/− SD.

The *APOE ε4* allele increases the risk of Alzheimer disease through multiple mechanisms, including an increase in amyloid plaques, CAA, and in plaque-mediated tangle accumulation^13-16^. The *APOE ε4* allele also increases ARIA risk in a dose-dependent manner, and carriers may also benefit less from lecanemab and donanemab compared to non-carriers. We explored the effect of *APOE ε4* gene dosage in our antibody binding data to help explain these phenomena. We found the *APOE ε4* allele lowered the parenchymal IS B_max_ ratio in a dose-dependent manner for all antibodies; in other words, the *APOE ε4/ε4* homozygotes possessed a more soluble pool of Aβ accessible to each anti-amyloid antibody compared to heterozygotes, in turn more soluble than non-carriers (Fig 4A). In meninges, homozygotes possessed a lower IS B_max_ ratio than heterozygotes and non-carriers, but there was no difference between heterozygotes and non-carriers (Fig 4B). There was no statistically significant effect of *APOE ε4* gene dosage on the IS K_D_ ratio in either parenchyma or in meninges; in other words, *APOE ε4* did not affect the affinity with which antibodies bound to soluble *vs.* insoluble Aβ, only their total availability to bind. We also detected no effect of *APOE ε4* dosage on either the absolute K_D_ to meningeal extracts or those normalized to parenchymal extracts (the MP K_D_ ratio); in other words, *APOE ε4* did not change Aβ conformation in such a way to significantly alter antibody affinity. Full model results are presented in the supplementary tables.

## Discussion

This study has important limitations. First, we only measured equilibrium binding constants (K_D_) as an approximation of affinity. We were unable to measure association (k_on_) and dissociation (k_off_) constants. Antibodies with identical equilibrium binding affinities can have different kinetics, and thus we cannot exclude that association and dissociation rates could differ among antibodies for parenchymal, vascular, soluble, or insoluble Aβ aggregates. We are developing techniques to measure these kinetics in AD brain extracts.

Second, the extraction method from the parenchyma could not separate plaque Aβ aggregates from microvascular CAA aggregates. Thus, some contamination in the parenchymal preparation with CAA aggregates was likely. However, we could enrich for plaque aggregates through the selective detection of Aβ_42_ over Aβ_40_ in the parenchymal fraction. Further, the meningeal preparations were unlikely to contain any plaque aggregates because amyloid plaques do not occur in the meninges. Thus, at best, the calculated MP ratios are of binding affinity to CAA *vs* plaque aggregates, and at worst, a ratio of binding affinity to CAA *vs* total (CAA + plaque) aggregates. This also assumes that CAA aggregates in meningeal blood vessels are biochemically equivalent to those in the parenchymal microvasculature, which they may not be. A last disadvantage of our approach is the exclusion of Aβ_40_-rich aggregates from the parenchyma; certain plaques are Aβ_40_-rich^17^, and antibody binding to these would not have contributed to our measures.

Third, we used antibodies produced recombinantly from patent sequences. While at least aducanumab and donanemab displayed similar binding in a subset of extracts (Fig S1), we cannot exclude subtle differences in binding compared to the brand-name product given to patients.

Fourth, we cannot exclude the extraction method itself causing structural alterations in the antibody epitopes. We have particular caution for interpreting donanemab results, because this is a non-conformational (linear) epitope. Homogenization may have sheared free pyroglutamate-3 Aβ monomers off of aggregates, leading to an excess of monomeric pyroglutamate-3 Aβ as opposed to that which naturally occurs in the aggregates. This artificially sheared pyroglutamate-3 Aβ could result in falsely low K_D_ and B_max_ values. Because aducanumab and lecanemab recognize conformational epitopes, shearing of monomers should not have caused this artifact, though we cannot exclude alterations in their epitope abundance during extraction.

Within these limitations, we conclude that 1) lecanemab does not bind a distinct population of Aβ aggregates inaccessible to aducanumab or donanemab, 2) that lecanemab exhibits equivalent binding affinity to “soluble protofibrils” (aqueously extracted Aβ aggregates) than aducanumab or donanemab, and 3) that the observation from phase 3 trials that lecanemab caused less ARIA-E than donanemab, in turn less than aducanumab, can be explained by differences in binding preference to CAA. In previous work, we found that anti-amyloid antibodies including lecanemab could bind to aqueously extracted short Aβ fibrils from human brain^18^, suggesting that much of what has previously been termed “protofibrils” and presumed to possess a different structure (and thus different antibody binding characteristics) compared to fibrils, are in fact structurally the same amyloid fibrils as those found in amyloid plaques. Our data from this study are consistent with this model: a putatively protofibril-preferring antibody (lecanemab) exhibited the same binding preference to the aqueous (“protofibrillar”) extraction as did the putatively non-protofibril-preferring antibody (aducanumab). Previous comparisons of lecanemab to other antibodies used *in vitro* synthetic preparations of Aβ^1^, the structures of which are unknown and may not exist in Alzheimer disease. One previous measurement comparing lecanemab to other antibodies’ binding to human meningeal extracts did not include a ratio to parenchymal extracts, tested only N=4 brains, and excluded one of these as an outlier^19^.

Our results do not exclude off-target CAA-binding as a cause of ARIA-E. Rather, we conclude only that *differences* between antibodies’ ARIA-E rates and efficacy cannot be explained by *differences* in the equilibrium binding constants we measured. A small dose titration adjustment in the Trailblazer-Alz 6 trial of donanemab reduced ARIA rates^20^, implying that at least some differences in ARIA-E rates to other antibodies could be explained by dosing or monitoring factors rather than differences in antibody affinity. The phase 3 trials for aducanumab, donanemab, and lecanemab differed in titration regimens and in the number of monitoring MRIs obtained; the latter could lead to sampling bias. While all three antibodies are IgG1 subclass, differences in Fc receptor engagement, half-life, off-rates or other antibody-dependent but binding equilibrium affinity-independent factors could additionally affect ARIA-E and efficacy.

We found that the *APOE ε4* allele was associated with an overall more soluble pool of Aβ that the antibodies could bind to. Future studies may relate how this alteration may affect therapeutic parameters such as clearance from the brain and neutralization of toxicity, and whether a more soluble pool of CAA or plaque Aβ results in an elevated risk of ARIA-E.

Future efforts to improve anti-amyloid therapy for Alzheimer disease will require a deeper mechanistic understanding of how antibody-antigen interactions shape plaque clearance, clinical efficacy, and ARIA.

## Supplementary Material

1

## Figures and Tables

**Table 1.** Case characteristics.

Case No.	Age (y)	Sex	*APOE* genotype	CERAD neuritic plaque score	Braak stage
**1**	73	F	ε3/ε4	1	6
2	68	F	ε4/ε4	2	4
3	66	F	ε3/ε4	2	5
4	75	M	ε3/ε4	1	4
5	73	F	ε3/ε4	1	5
6	70	F	ε4/ε4	2	6
7	79	M	ε3/ε3	1	3
8	73	M	ε3/ε4	2	5-6
9	73	M	ε3/ε4	1	5-6
10	82	M	ε3/ε4	1	3
11	68	F	ε4/ε4	2	6
12	65	F	ε3/ε4	2	5-6
13	88	M	ε3/ε3	2	5-6
14	74	M	ε3/ε3	1	5
15	79	M	ε4/ε4	2	5
16	75	M	ε2/ε4	0	0
17	80	F	ε3/ε4	1	3
18	77	F	ε4/ε4	1	5
