# Supplementary material for: Anti-amyloid antibody equilibrium binding to Aβ aggregates from human Alzheimer disease brain
Source: bioRxiv. 2025 May 21:2025.05.20.654902. Preprint. [Version 1] doi: 10.1101/2025.05.20.654902 (PMC12139811; doi:10.1101/2025.05.20.654902)
Supplement: 1 [file NIHPP2025.05.20.654902v1-supplement-1.pdf]

bioRxiv preprint doi: https://doi.org/10.1101/2025.05.20.654902; this version posted May 21, 2025. The copyright holder for this preprint (which was not certified by peer review) is the author/funder, who has granted bioRxiv a license to display the preprint in perpetuity. It is made available under aCC-BY 4.0 International license.

## Supplementary Figures

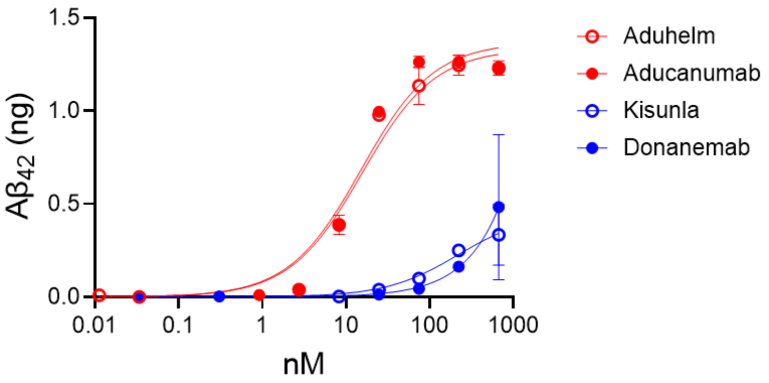


**Fig S1.** Binding profile of recombinant aducanumab and donanemab equivalents used for this study were identical to those of their brand-name products.

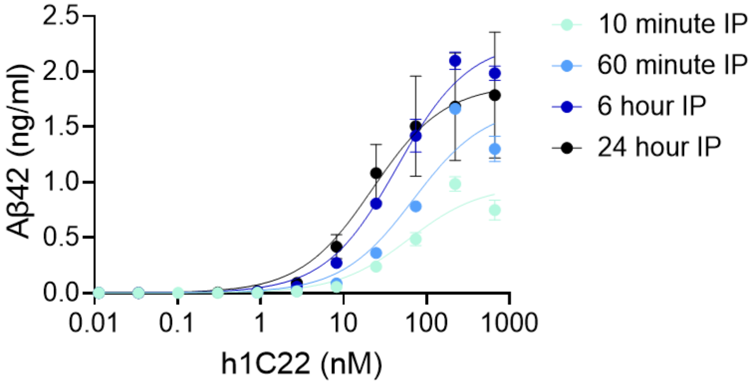


**Fig S2.** Past 6 hours, the binding curve for test antibody h1C22 equilibrated. Thus, we used a 24-hour immunoprecipitation reaction to measure equilibrium binding of anti-amyloid antibodies.

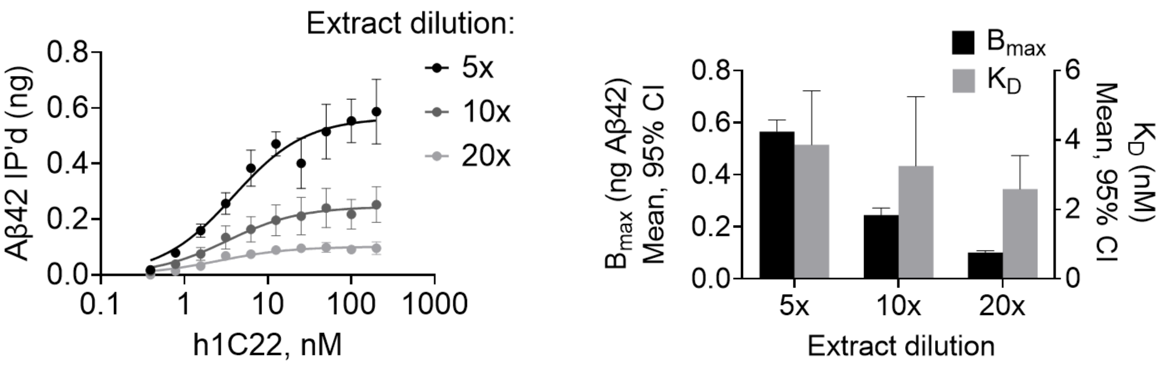

bioRxiv preprint doi: https://doi.org/10.1101/2025.05.20.654902; this version posted May 21, 2025. The copyright holder for this preprint (which was not certified by peer review) is the author/funder, who has granted bioRxiv a license to display the preprint in perpetuity. It is made available under aCC-BY 4.0 International license.

**Fig S3.** As a human AD brain extracted is diluted 5-, 10-, or 20-fold, the $B_{max}$ diminishes proportionately to the dilution factor but the $K_D$ does not, implying a measurement of antibody affinity rather than antibody titration.

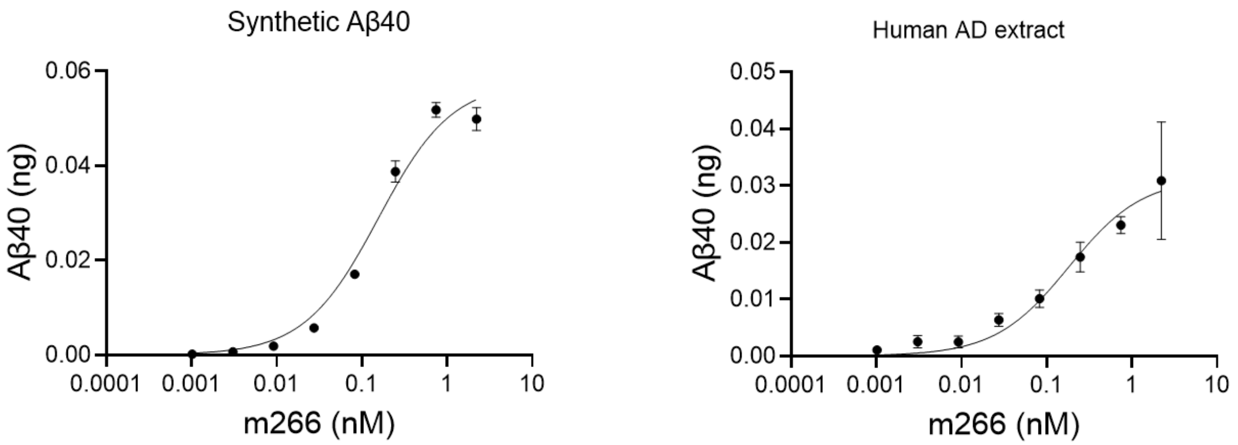


**Fig S4.** The binding of test antibody m266, which binds the Aβ mid-region and selectively binds monomers, exhibits the same binding profile with synthetic $A\beta_{40}$ monomers as those found naturally in a human AD extract. This antibody was chosen to test the validity of the method because the m266 epitope, being linear and present on monomers, is expected to have the same structure in synthetic as human brain-derived extracts.

## <u>Supplementary Methods</u>

### Extraction of Aβ aggregates from parenchyma and meninges

We optimized a protocol based on multiple literature sources[21–23] for the extraction of aqueous and insoluble Aβ aggregates from both grey matter and meninges. First, an occipital coronal slice of frozen brain tissue was thawed on ice. The leptomeninges were peeled off with

bioRxiv preprint doi: https://doi.org/10.1101/2025.05.20.654902; this version posted May 21, 2025. The copyright holder for this preprint (which was not certified by peer review) is the author/funder, who has granted bioRxiv a license to display the preprint in perpetuity. It is made available under aCC-BY 4.0 International license.

forceps and then chopped on a McIlwain tissue chopper set to 0.05 mM. The grey matter was dissected from the white matter with a scalpel. Both the grey matter and meninges were weighed and resuspended five volumes TBS extraction buffer containing 25 mM Tris, 150 mM NaCl, pH 7.4 supplemented with 5 µg/ml leupeptin, 5 µg/ml aprotinin, 2 µg/ml pepstatin, 120 µg/ml 4-benzenesulfonyl fluoride hydrochloride, and 5 mM NaF. Both were homogenized using a Teflon Dounce homogenizer attached to an IKA stirrer at 800 rpm for 25 strokes. The parenchymal homogenate, being of greater volume, was centrifuged in a Type 70.1Ti rotor (Beckman) in thickwall polycarbonate tubes (Beckman cat. 355630) at 25,000 rpm ($rcf_{av}$ = 42,800 *g*) for 20 min at 4°C. The meningeal homogenate was centrifuged in 1.5-ml microcentrifuge tubes in a tabletop centrifuge at 20,000 *g* for 2 hours. We found that these two centrifugation protocols, adapted to different volumes, resulted in similar amounts of Aβ in the supernatant for the same sample. The top 90% supernatant was retained as the aqueously extracted Aβ fraction, and the bottom 10% discarded. The pellets were frozen at -80°C for later processing.

To extract insoluble Aβ from parenchyma, the parenchymal pellet was thawed, re-weighed, and resuspended in five volumes TBS extraction buffer supplemented with 2% N-lauryl sarcosine (sarkosyl). The suspension was incubated at 37°C for 1 hour, then re-homogenized as above. The homogenate was centrifuged in a TLA-55 rotor at 55,000 rpm ($rcf_{av}$ = 136,000 x *g*) for 1 hour at 4°C. The pellet was washed twice in 1 ml TBS without sarkosyl by resuspending and centrifuging again in a TLA-55 rotor at 55,000 rpm ($rcf_{av}$ = 136,000 x *g*) for 1 hour at 4°C. The final pellet was weighed once more and resuspended in five volumes TBS, then sonicated for 25 seconds at 35% power prior to aliquoting. After thawing and dilution for IP-ELISA, the extract was sonicated again for 5 minutes at 35% power.

To generate insoluble Aβ from meninges, we followed a protocol used in the literature to derive meningeal fibrils[22,24]. The meningeal pellet from the aqueous extraction was thawed and sliced again with a scalpel. The sliced material was weighed again and washed three times in

bioRxiv preprint doi: https://doi.org/10.1101/2025.05.20.654902; this version posted May 21, 2025. The copyright holder for this preprint (which was not certified by peer review) is the author/funder, who has granted bioRxiv a license to display the preprint in perpetuity. It is made available under aCC-BY 4.0 International license.

five volumes Tris-calcium buffer containing 20 mM Tris, pH 8.0 with 138 mM NaCl, 2 mM $CaCl_2$, and 0.1% $NaN_3$ followed by centrifuging at 20,000 *g* for 5 minutes in a tabletop centrifuge. The washed pellet was then resuspended once more in Tris-calcium buffer supplemented with 5 mg/ml collagenase from *Clostridium histolyticum* (Sigma) and nutated overnight at 37°C. Remaining steps were carried out on ice or at 4°C. The suspension was centrifuged at 20,000 *g* for 10 minutes in a tabletop centrifuge. The pellet was then washed twice by resuspending in two volumes wash buffer containing 50 mM Tris, 10 mM EDTA, pH 8.0 and centrifuging in a TLA-55 rotor at 55,000 rpm ($rcf_{av}$ = 136,000 x *g* ). The pellet was resuspended in one volume deionized water and homogenized with a hand-held homogenizer (Fisher), then centrifuged at 12,000 *g* for 5 minutes. The supernatant was saved, and the pellet was again resuspended in water and centrifuged again at 12,000 *g* for 5 minutes. This water extraction was performed five times, and all five fractions were pooled and then aliquoted for IP-ELISA.

**Antibody sources**

Recombinant human aducanumab, lecanemab, and donanemab were produced recombinantly by Evitria, Inc. in Chinese Hamster Ovary (CHO) cells according to the amino acid sequence found in the patent literature. Antibody purity was verified by HPLC. Mouse antibodies 266, 2G3 and 21F12 were gifts from Elan Pharmaceuticals. Aducanumab-avwa (Aduhelm) and donanemab-azbt (Kisunla) for verification experiments were purchased from McKesson.

**Measurement of antibody binding to Aβ aggregates from human brain**

Brain extracts were diluted in TBS-T (25 mM Tris pH 7.4, 500 mM NaCl, 0.05% Tween-20) in the presence of 20 µl washed protein G beads (Bio-Rad) and a three-fold dilution series of the IP antibody starting at 100 µg/ml (666.67 nM) in 96-well round-bottom protein low-binding plates (Greiner), in a final volume of 150 µl. To approximate the same concentrations of antigen,

bioRxiv preprint doi: https://doi.org/10.1101/2025.05.20.654902; this version posted May 21, 2025. The copyright holder for this preprint (which was not certified by peer review) is the author/funder, who has granted bioRxiv a license to display the preprint in perpetuity. It is made available under aCC-BY 4.0 International license.

aqueous extracts were diluted 10-fold, insoluble parenchymal extracts were diluted 2,000-fold, and insoluble meningeal extracts were diluted 200-fold. The IP reactions were carried out at 4°C for 18 hours on a plate shaker at 800 rpm. The beads were washed three times in 200 µl TBS-T followed by elution in 50 µl 5 M GuHCl shaking overnight at 4°C. The eluate was then quantified by MSD Aβ ELISAs (see method below). The Aβ42 assay was used to quantify the parenchymal IP, and the Aβ40 assay to quantify the meningeal IP. The IP antibody concentration in nM was normalized to the background (0 nM antibody) by subtraction and plotted against the amount of Aβ IP'd. The data were fitted to a one-site specific binding model (GraphPad Prism) of the form $A\beta = B_{max}*X/(K_D + X)$, where X represents the IP antibody concentration. Fitting was accomplished by least squares regression without weighting or constraints. We regard the $K_D$ as an approximate measure of binding affinity, and the $B_{max}$ as a measure of the total Aβ available for binding by the test antibody. The $K_D$ is not a precise measure of affinity, however, because the molar quantity of Aβ aggregates is unknown – only the concentration of total monomers composing those aggregates is quantitated. However, by comparing all antibodies across all brains, we can make relative comparisons between antibodies, even if the $K_D$ is not an exact measure of affinity.

We used the excellent paper by Jarmoskaite *et al.*[25] as a guide to ensure we measured binding properties of the antibodies of interest. We validated the method using a humanized version of test antibody 1C22, for which *in vitro* binding characteristics had previously been evaluated[26]. Past six hours of incubation time, the binding curves had saturated (Fig S2), indicating the reaction was at equilibrium – we chose an 18-hour binding time going forward for convenience. We serially diluted the brain extracts and evaluated the binding curves (Fig S3), finding that the $B_{max}$ was proportionately lowered as expected, but the $K_D$, was not, indicating that the $K_D$ is measuring a binding property of the antibody-antigen interaction, rather than a simple titration of the antibody out of solution as would be the case if the antigen concentration were too high[25].

bioRxiv preprint doi: https://doi.org/10.1101/2025.05.20.654902; this version posted May 21, 2025. The copyright holder for this preprint (which was not certified by peer review) is the author/funder, who has granted bioRxiv a license to display the preprint in perpetuity. It is made available under aCC-BY 4.0 International license.

We tested whether the IP-ELISA method estimated the binding affinities similarly in complex brain extracts as in pure solution. Using IP antibody m266, which has strong preference for monomers, we evaluated its $K_D$ in pure solution for Aβ40 (Fig S4) at approximately 23 nM, which agreed with the value for a meningeal AD extract at 22 nM.

We were able to purchase brand-name Aduhelm (aducanumab-avwa) and Kisunla (donanemab-azbt), and compare these binding properties to the recombinant aducanumab and donanemab used in this study (Fig S1), showing no significant difference.

**MSD ELISAs**

Aβ monomer-preferring ELISAs were home-brew immunoassays as described[18]. Samples containing GuHCl were diluted such that the GuHCl concentration was ≤0.25 M to avoid interference with the antibody reaction. All steps were at room temperature. Plates were coated with capture antibody 266 at 3 µg/ml in PBS overnight and blocked in 5% MSD Blocker A in TBS with 0.05% Tween-20 (TBS-T) for 1.5 h. Samples were then applied for 1.5 h after being diluted in 1% Blocker A in TBS-T. Plates were washed 3 times in TBS-T, then biotinylated detector antibody and MSD Strepavidin-Sulfotag (1:5,000) were applied for 1.5 h. Biotinylated antibodies 21F12 at 0.4 µg/ml and 2G3 at 0.2 µg/ml were used as detectors for the Aβ42 and Aβ40 assays, respectively. After three more washes in TBS-T, the plates were detected with 2x MSD read buffer T. The lower limit of quantification (LLoQ) was defined as the lowest standard with a luminescence value at least twice the blank average, and the lower limit of detection (LLoD) was defined as the lowest standard with values greater than the blank average plus twice the blank standard deviation.

bioRxiv preprint doi: https://doi.org/10.1101/2025.05.20.654902; this version posted May 21, 2025. The copyright holder for this preprint (which was not certified by peer review) is the author/funder, who has granted bioRxiv a license to display the preprint in perpetuity. It is made available under aCC-BY 4.0 International license.

## Supplementary Tables

| | Estimate | Std. Error | df | t value | Pr(>\|t\|) |
|---|---|---|---|---|---|
| (Intercept) | -0.17825 | 0.879401 | 14.07809 | -0.20269 | 0.842274 |
| Aducanumab | 0.023238 | 0.080296 | 34 | 0.289406 | 0.774028 |
| Donanemab | -0.22528 | 0.080296 | 34 | -2.80563 | 0.008248 |
| APOE (# ε4) | 0.193307 | 0.090766 | 14 | 2.129725 | 0.05142 |
| Age | -0.00352 | 0.012026 | 14 | -0.29284 | 0.773939 |
| Female sex | 0.321972 | 0.144682 | 14 | 2.225369 | 0.043003 |

**Table S1**. Fixed effects of linear mixed model for parenchymal log(IS $K_D$ ratio).

| | Estimate | Std. Error | df | t value | Pr(>\|t\|) |
|---|---|---|---|---|---|
| (Intercept) | -0.49904 | 1.083065 | 14.05464 | -0.46077 | 0.652014 |
| Aducanumab | -0.0879 | 0.082765 | 34 | -1.06207 | 0.295689 |
| Donanemab | -0.05415 | 0.082765 | 34 | -0.65432 | 0.517308 |
| APOE (# ε4) | 0.222131 | 0.111834 | 14 | 1.986261 | 0.066942 |
| Age | 0.006821 | 0.014817 | 14 | 0.460373 | 0.652318 |
| Female sex | -0.00129 | 0.178264 | 14 | -0.00725 | 0.994314 |

**Table S2**. Fixed effects of linear mixed model for soluble log(MP $K_D$ ratio).

| | Estimate | Std. Error | df | t value | Pr(>\|t\|) |
|---|---|---|---|---|---|
| (Intercept) | 0.145335 | 1.043693 | 14.03173 | 0.139251 | 0.891231 |
| Aducanumab | -0.16181 | 0.060808 | 34 | -2.66098 | 0.011806 |
| Donanemab | 0.26036 | 0.060808 | 34 | 4.281659 | 0.000143 |
| APOE (# ε4) | -0.04043 | 0.107812 | 14 | -0.37499 | 0.713285 |
| Age | 0.001281 | 0.014284 | 14 | 0.089665 | 0.929824 |
| Female sex | -0.16735 | 0.171854 | 14 | -0.97377 | 0.346697 |

**Table S3**. Fixed effects of linear mixed model for insoluble log(MP $K_D$ ratio).

| | Estimate | Std. Error | df | t value | Pr(>\|t\|) |
|---|---|---|---|---|---|
| (Intercept) | 2.641361 | 1.807181 | 14.00999 | 1.461592 | 0.165919 |
| Aducanumab | 0.000952 | 0.059124 | 34 | 0.016098 | 0.98725 |
| Donanemab | -0.0935 | 0.059124 | 34 | -1.58134 | 0.123059 |
| APOE (# ε4) | -0.57583 | 0.186752 | 14 | -3.08337 | 0.008095 |
| Age | -0.03679 | 0.024743 | 14 | -1.48674 | 0.159255 |
| Female sex | -0.22632 | 0.297685 | 14 | -0.76026 | 0.459712 |

**Table S4**. Fixed effects of linear mixed model for parenchymal log(IS $B_{max}$ ratio).

bioRxiv preprint doi: https://doi.org/10.1101/2025.05.20.654902; this version posted May 21, 2025. The copyright holder for this preprint (which was not certified by peer review) is the author/funder, who has granted bioRxiv a license to display the preprint in perpetuity. It is made available under aCC-BY 4.0 International license.
